# Pets, furry animal allergen components, and asthma in childhood

**DOI:** 10.1002/clt2.12337

**Published:** 2024-02-05

**Authors:** Katariina Lajunen, Anette M. Määttä, Kristiina Malmström, Satu Kalliola, Hanna Knihtilä, Terhi Savinko, L. Pekka Malmberg, Anna S. Pelkonen, Mika J. Mäkelä

**Affiliations:** ^1^ Department of Allergology Skin and Allergy Hospital University of Helsinki and Helsinki University Hospital Helsinki Finland; ^2^ Department of Paediatrics and Adolescents Turku University Hospital Turku Finland; ^3^ Department of Pediatrics Stanford University School of Medicine Stanford California USA

**Keywords:** allergen component, asthma, eosinophilia, furry animal, pets

## Abstract

**Background:**

The use of component‐resolved allergy diagnostics has provided a clearer understanding of the species‐specific sensitization and severity of potential allergic reactions. This cross‐sectional study aimed to determine whether sensitization to allergen components in furry animals is indicative of blood eosinophilia, a positive fractional exhaled nitric oxide (FeNO) test, abnormal lung function, and asthma symptoms in children. Additionally, we investigated whether having pets during childhood affects the development of asthma or allergic sensitization to furry animals.

**Methods:**

We recruited 203 children aged 4–17 years with asthma diagnosis based on abnormal lung function and 33 controls. IgE‐sensitization to allergen components for dogs, cats, and horses was analyzed using a multiplex microarray. Children were tested with FeNO, impulse oscillometry, spirometry, methacholine challenge, and skin prick test. A questionnaire was used to investigate pet ownership and symptom profile.

**Results:**

FeNO results and blood eosinophilia revealed a correlation with sensitization to all furry animal allergens, particularly lipocalins (*r* = 0.203–0.560 and 0.206–0.560, respectively). Can f 3 was found to correlate with baseline R5 (*r* = 0.298). No association between methacholine challenge results and sensitization to furry animal allergens was found. Children with asthma who were sensitized to Can f 1, Can f 6, or both frequently reported asthma symptoms. Dog ownership was associated with a lower level of IgE‐sensitization to lipocalins, fewer asthma symptoms, and less blood eosinophilia.

**Conclusion:**

Furry animal allergen component IgE‐sensitization is a risk factor for type 2‐inflammation and asthma symptoms.

## 1 INTRODUCTION

The prevalence of sensitization to furry animals has increased worldwide over the past 3 decades.^1^, ^2^, ^3^, ^4^ Although skin prick tests (SPTs) with standardized animal extracts are recommended as the initial diagnostic procedure in patients with clinically suspected furry animal allergy,^3^ allergen components offer the possibility of defining sensitization to individual allergenic molecules and can clarify the meaning of those specific sensitization with regard to the intensity of allergic symptoms and cross‐reactivity.^4^

Animal serum albumins such as Can f 3, Equ c 3, and Fel d 2 are considered to be minor allergens and highly cross‐reactive.^4^, ^5^, ^6^ In contrast, lipocalins such as Can f 1 and 6, Fel d 4, and Equ c 1 together with uteroglobin Fel d 1 are considered as major allergens. Sensitization of furry animals to allergens is an asthma risk.^6^, ^7^, ^8^

According to an adult study, allergen component sensitization in general have higher odds ratios for current asthma than allergen extracts.^7^ Patients with rhinitis and sensitization to furry animal allergens more often had asthma than their non‐sensitized counterparts.^8^ The co‐morbidity was particularly pronounced with major allergens. Multi‐sensitization to Fel d 4 and Can f 1 was associated with more severe asthma in children.^9^ This may be due to increased airway hyper‐responsiveness (AHR), which has been linked to positive SPTs to cats and dog.^7^, ^10^, ^11^ In severe childhood asthma, multi‐sensitization toward major furry animal allergens is also associated with bronchial inflammation measured using fractional exhaled nitric oxide (FeNO) and blood eosinophilia.^12^ In other studies, several furry animal allergen sensitization were associated with bronchial inflammation and AHR but not with blood eosinophilia^7^, ^8^ or spirometry results.^13^

We aimed to investigate whether allergen component IgE‐sensitization to furry animals predicts more pronounced type 2 inflammation reaction and asthma symptoms in children. Our secondary aim was to assess whether having pets during childhood impacts the development of asthma or allergic sensitization to furry animals.

## 2 METHODS

**FIGURE 1:**
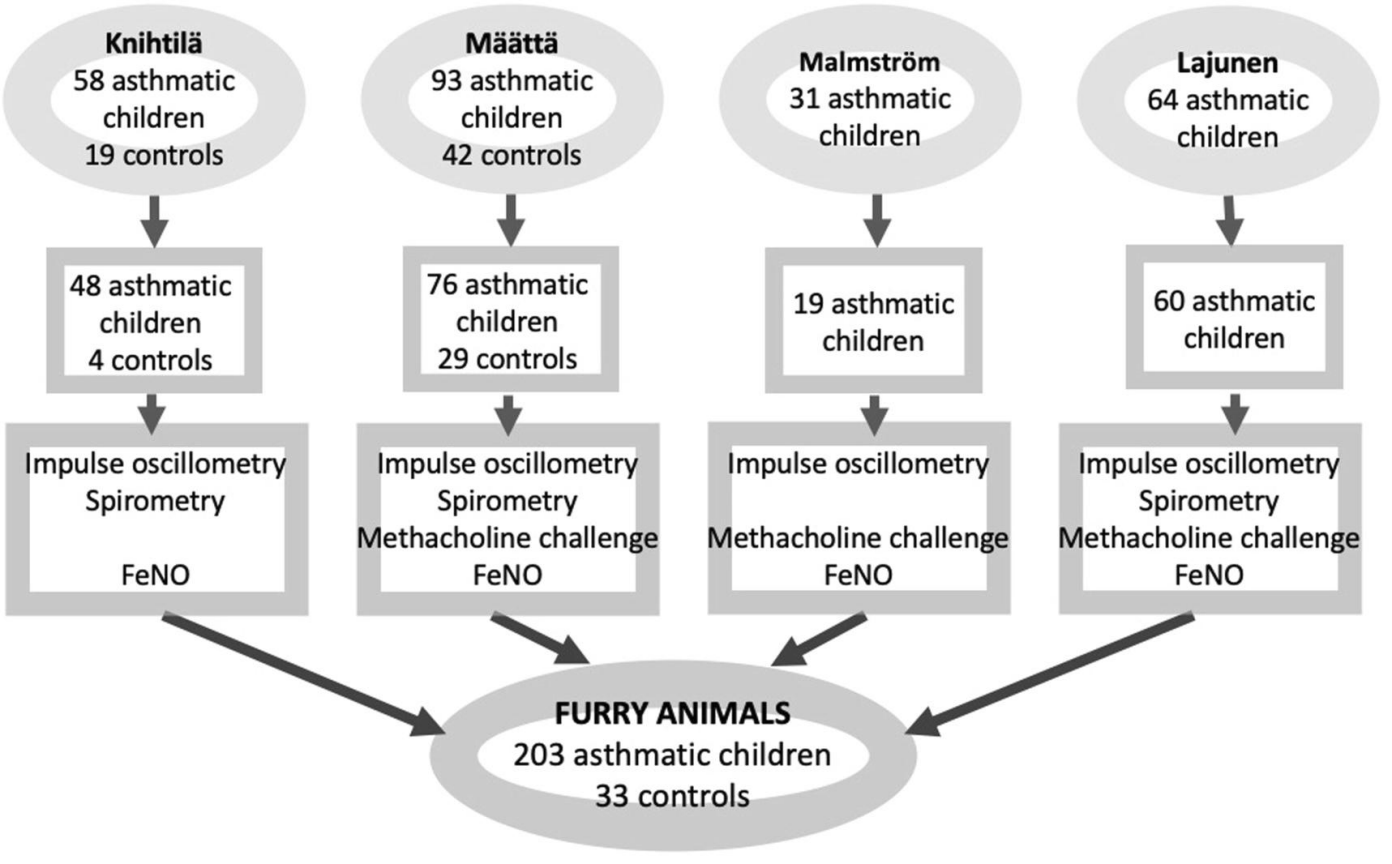
The flowchart of participants and lung function measurements of all the four studies^14^, ^15^, ^16^, ^17^ included. FeNO, fractional exhaled nitric oxide.

We recruited 203 Finnish children aged 4–17 years with an asthma diagnosis based on abnormal lung function and 33 controls from four different studies^14^, ^15^, ^16^, ^17^ in Skin and Allergy Hospital, Helsinki, Finland between 2005 and 2014. A flowchart of study children and lung function measures included in the respective study protocols is shown in Figure 1. Blood serum was collected, and pulmonary tests were performed within 4 months. The protocol excluded children who used systemic or inhaled corticosteroids, leukotriene receptor antagonists >1 month prior, and bronchodilators 12 h prior, and those with respiratory infection symptoms within 2 weeks prior to lung function measures.

The participants or parents completed a detailed questionnaire. This self‐report included asthma‐like symptoms such as wheezing, dyspnea, rhonchi, and persistent cough during the last 12 months. This study was conducted according to the principles of the Declaration of Helsinki. All the four studies^14^, ^15^, ^16^, ^17^ included were approved by the Research Ethics Committee of the Helsinki University Central Hospital (139/13/03/03/2011, 416/E7/2003, 390/13/03/03/2015, and 211/13/03/03/2015, respectively). All study children and their parents provided written informed consent before enrollment.

SPTs were performed using standardized extracts and were considered positive if a child had a wheal diameter of ≥3 mm. The following 10 local aeroallergens were tested: birch, timothy grass, meadow fescue, mugwort, *Cladosporium herbarum*, dog, cat, horse, cow, and house dust mite. A blood eosinophil percentage ≥4% and/or blood eosinophil count ≥470 cells/mL was interpreted as eosinophilia.

### 2.1 Allergen component sensitization testing

An ISAC multiplex microarray (ISAC Chip, Thermo Fisher Scientific) was used to analyze IgE‐sensitization to 112 different allergen components.^18^ A cut‐off level of ≥0.3 ISU (ISAC standardized units) was considered positive. We chose certain molecule‐specific allergens of dog (Can f 1, Can f 2, Can f 3, Can f 5, and Can f 6), cat (Fel d 1, Fel d 2, and Fel d 4), and horse (Equ c 1 and Equ c 3) for further analysis.

The ImmunoCAP ISAC chips were washed and dried before use. The chip was covered with 30 μL of serum followed by 120 min incubation in a humidity chamber. After incubation, the chips were rinsed, washed, and dried. The chips were then covered with 30 μL IgE detection antibody solution and incubated at room temperature using the humidity chamber for 30 min. After the second incubation, the chips were rinsed, washed, and dried. The chip was scanned with a laser scanner (LuxScan 10 K/A, Capitol‐Bio) and analyzed using Phadia MIA Software.

### 2.2 Pulmonary function

All children performed impulse oscillometry and 205 children performed spirometry after a minimum of 1 month of discontinuation of anti‐inflammatory asthma medication. Oscillometry was performed using an IOS apparatus (Jaeger GmbH). Spirometry was performed using a pneumotachograph‐based spirometer (Masterscreen Pneumo, Carefusion) according to ATS recommendations.^19^ All measurements were combined with a bronchodilation test (BDT). Children aged <10 years inhaled 300 μg albuterol (Ventoline, GSK) via Babyhaler and children ≥10 years inhaled 400 μg via Volumatic. BDT was considered positive if inhalation resulted in ≥40% decrease in respiratory resistance at 5 Hz (R5) or ≥12% increase in forced expiratory volume in one second (FEV1).^20^

Methacholine challenge tests were performed by 183 children using an automatic breath actuating dosimeter (Spira Elektro 2) with spirometry or IOS for those aged <7 years. The protocol included five cumulative dose steps of inhaled methacholine. Using a dose‐response curve, the provocative dose produces a ≥20% fall in FEV (PD_20_FEV1) or a ≥40% increase in R5 (PD_40_R5) was determined.^21^ The test was considered positive for AHR if PD_20_FEV1 or PD_40_R5 was ≤400 μg.^22^

FeNO was assessed in paired measurements with a chemiluminescence analyzer (NIOX, Aerocrine AB or CLD 88, Eco Medics) according to the current recommendations.^23^ The test protocol consisted of exhaling air at a constant flow of 50 mL/s for ≥6 s, including a plateau phase of ≥2 s. The test was considered positive with a *z*‐score ≥2.0.^24^

### 2.3 Statistics

According to power calculations (Supporting Information S1) a study population exceeding 62 children and 32 or more control children will provide a significant result with 95% confidence and accepting a beta cut‐off of 20%. Correlations were calculated using the Spearman 2‐tailed correlation test and independent continuous data of more than two groups with One‐way ANOVA combined with covariate analysis with ANCOVA. *χ* ^2^ test was used to analyze categorical data and linear regression analysis to calculate odd’s ratios (OR). A general linear univariate model together with covariate analysis, including age and gender as covariates, was used for multiple variable analysis. Data were analyzed using IBM SPSS 27.0 (Armonk).

## 3 RESULTS

The study groups had similar demographic characteristics (Table 1). The asthmatic children had a higher frequency of asthma‐like symptoms, atopy, positive FeNO, abnormal lung function, and a higher total IgE level than the controls. However, the prevalence of AHR to methacholine in both groups was surprisingly similar. The households of asthmatic children more likely had pets and these children more likely were exposed to environmental tobacco smoke (ETS) than the controls, although the difference in ETS was not statistically significant.

**TABLE 1:** Demographics.

	Asthmatics	Controls	*p*‐value
*n*	203	33	
Female, %	31	42	0.183
Birthweight, kg [range]	3.56 [1.5:4.9]	3.68 [2.8:4.6]	0.215
Age, yrs [range]	11.0 [4.5:17.1]	10.0 [5.3:13.0]	0.101
Height, cm (SD)	145.8 (18.9)	140.7 (11.7)	0.139
Weight, kg (SD)	41.9 (16.4)	35.3 (9.8)	0.026
Exposure to environmental smoking, *n* (%)	52 (26)	4 (12)	0.092
Pets, *n* (%)	106 (52)	9 (27)	0.007
Asthma symptoms during last year,^a^ *n* (%)	147 (72)	4 (12)	<0.001
Atopy,^b^ *n* (%)	129 (64)	13 (39)	0.009
Blood eosinophilia, ≥4%, *n* (%)	94 (46)	15/30 (50)	0.724
Blood eosinophilia, ≥470 cells/mL, *n* (%)	58 (29)	10/30 (30)	0.669
Total serum IgE, kU/l [range]	152 [2.0:24,330.0]	92 [6.0:1100.0]	0.193
PD_20_FEV1 ≤400 μg, *n* (%)	59/153 (39)	10/29 (34)	0.708
FeNO ≥2SD, *n* (%)	67/198 (34)	3/31 (10)	0.006
BDT in IOS, R5 ≤‐40%, *n* (%)	6 (3)	0	0.324
BDT in spirometry, FEV1 ≥12%, *n* (%)	23/180 (13)	0	0.030

Abbreviations: BDT, bronchodilation test; FeNO, fractional concentration of exhaled nitric oxide; FEV1, forced expiratory volume in 1 s; IOS, impulse oscillometry; PD_20_FEV1, the provocative dose of inhaled methacholine producing a ≥20% fall in FEV; R5, respiratory resistance at 5 Hz.

^a^ Self‐reported from previous year.

^b^ Atopy is defined primarily by positive skin prick test and secondly by sensitization to allergen components of a local allergen selection.

### 3.1 FeNO and blood eosinophilia

**FIGURE 2:**
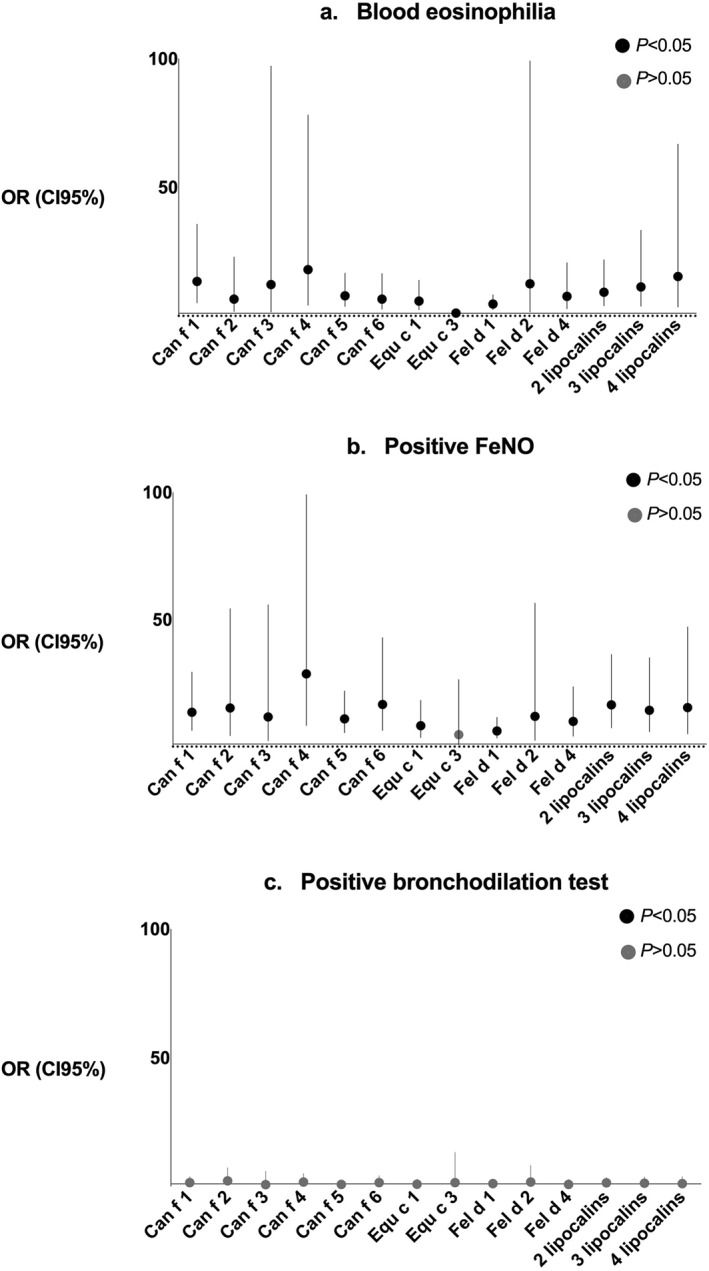
(A–C) The odds ratio with 95% confidence interval between (A) blood eosinophilia (%), (B) FeNO, and (C) lung function measurements and >0.3 ISAC‐standardized units sensitization to individual IgE components of furry animals. Blood eosinophil count ≥4% was considered eosinophilia. ≥40% increase in R5 in IOS, ≥12% decrease in FEV1 in spirometria, or both were interpreted as positive BDT. FeNO *z*‐score ≥2 SD was considered positive. BDT, bronchodilation test; CI, confidence interval; FeNO, fractional concentration of exhaled nitric oxide; IOS, impulse oscillometry.

**FIGURE 3:**
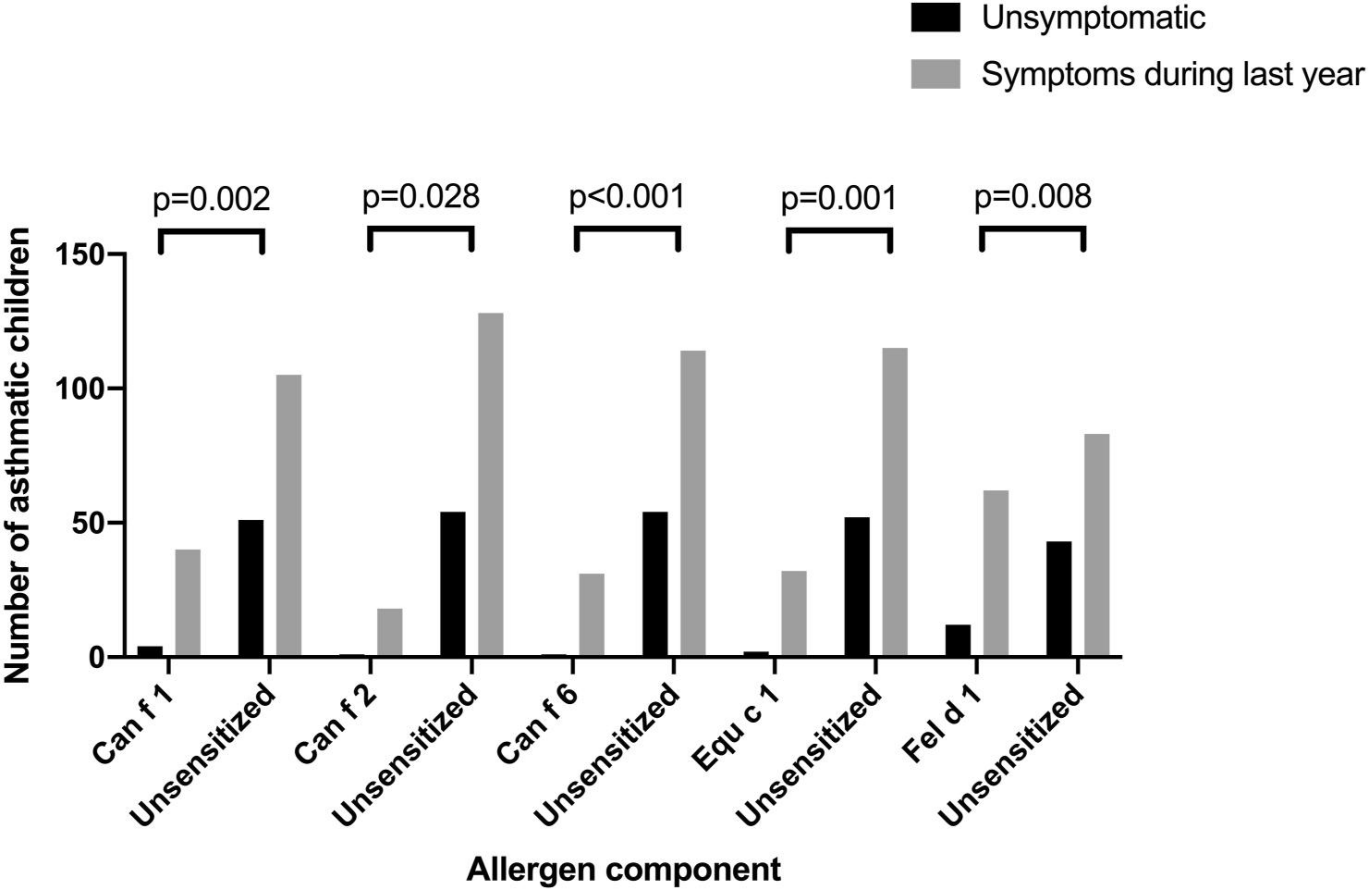
Connection between asthma symptoms over the last year and sensitization (>0.3 ISAC standardized units) to individual IgE components of furry animals.

Blood eosinophilia and positive FeNO were associated with each other (51% sensitivity and 88% specificity, *p* < 0.001). Blood eosinophilia and positive FeNO test correlated with sensitization to all furry animal allergens among asthmatic children, primarily with lipocalins and uteroglobin (Table 2), but not among control children (data not shown). Similarly, both blood eosinophilia and FeNO results were closely associated with sensitization to furry animal allergens, particularly lipocalins (Figure 2A–C), and with a high prevalence of asthma‐like symptoms (*p* < 0.001). Children sensitized to lipocalins were seven times more likely to report asthma‐like symptoms (OR = 7.0, 95% confidence interval [CI] 3.0:16.3) than those sensitized to other furry animal components (Figure 3). Lipocalins were also the most significant IgE‐components in distinguishing control children from those with asthma (Table 3).

**TABLE 2:** Spearman correlation coefficient (*r*) between sensitization furry animal allergen components and positive FeNO test, blood eosinophilia, and furry animal SPT results of asthmatic children.

			Eosinophilia		Positive SPT		
Component		FeNO	(≥4%)	(≥470 cells/mL)	Dog	Cat	Horse
Can f 1	Lipocalin	0.492	0.434	0.335	0.547	0.488	0.564
Can f 2	Lipocalin	0.343	0.233	0.266	0.325	0.351	0.485
Can f 3	Serum albumin	0.247	0.213	0.232	0.272	0.291	0.432
Can f 4	Lipocalin	0.472	0.363	0.264	0.339	0.438	0.496
Can f 5	Prostatic kallikrein	0.494	0.431	0.412	0.603	0.525	0.516
Can f 6	Lipocalin	0.447	0.305	0.262	0.485	0.530	0.617
Fel d 1	Uteroglobin	0.420	0.324	0.294	0.585	0.942	0.583
Fel d 2	Serum albumin	0.249	0.217	0.239	0.275	0.295	0.440
Fel d 4	Lipocalin	0.368	0.319	0.308	0.404	0.489	0.766
Equ c 1	Lipocalin	0.378	0.300	0.274	0.534	0.574	0.917
Equ c 3	Serum albumin	0.121	0.189	0.211	0.173	0.186	0.279

*Note*: Limits for a positive result are ≥2 SD for FeNO, ≥4% and ≥470 cells/mL for blood eosinophilia, >0.3 ISU for allergen component sensitization, and a wheal diameter of ≥3 mm for SPT. Adjusted for age, sex and environmental tobacco smoke. All results presented are significant, *p* < 0.05.

Abbreviations: FeNO, fractional exhaled nitric oxide; SPT, skin prick test.

**TABLE 3:** The number of sensitized children.

Allergen	Content	Asthmatics, %		Controls, %		*p*‐value
		Dog ownership	No dog	Dog ownership	No dog	
Dog SPT	STD dog extract	34	44	0	16	0.010
Can f 1	Lipocalin	10	27	0	7	0.034
Can f 2	Lipocalin	5	11	0	0	0.067
Can f 3	Serum albumin	6	4	0	0	0.171
Can f 4	Lipocalin	12	15	0	0	0.023
Can f 5	Prostatic kallikrein	23	29	0	4	0.002
Can f 6	Lipocalin	17	15	0	4	0.049
Any		28	38	0	11	0.003

		Cat ownership	No cat	Cat ownership	No cat	
Cat SPT	STD cat extract	36	38	0	11	0.005
Fel d 1	Uteroglobin	37	37	0	25	0.081
Fel d 2	Serum albumin	15	4	0	0	0.169
Fel d 4	Lipocalin	26	13	0	4	0.058
Any		41	37	0	25	0.071

		Pet ownership	No pet	Pet ownership	No pet	
Horse SPT	STD horse extract	17	25	0	12	0.160
Equ c 1	Lipocalin	14	20	0	4	0.039
Equ c 3	Serum albumin	5	1	0	0	0.318
Any		17	20	0	4	0.035
2 Lipocalins		22	22	0	8	0.003
3 Lipocalins		16	17	0	4	0.001
4 Lipocalins		11	12	0	0	<0.001

*Note*: Limits for a positive result are >0.3 ISU for allergen component sensitization and a wheal diameter of ≥3 mm for SPT. *p*‐value computed using *χ* ^2^ test in asthmatics (*n* = 203) and controls (*n* = 33).

Abbreviations: ISU, ISAC standardized units, SPT, skin prick test, STD, standardized.

### 3.2 Lung function

Only 7% of children diagnosed with asthma based on lung function measures showed positive BDT after discontinuation of anti‐inflammatory asthma medication. The children with a positive BDT were rarely sensitized to furry animal allergen components (Figure 2A–C). Furthermore, they reported asthma‐like symptoms significantly less than the children sensitized to furry animals (*p* = 0.209 vs. *p* < 0.001). After adjusting for covariates, Can f 3 was the only allergen component (*r* = 0.298) that correlated with baseline respiratory resistance at 5 Hz (R5) *z*‐score. No correlation was found between allergen component sensitization and baseline FEV1 *z*‐scores.

Although an initial correlation was detected between AHR and methacholine and sensitization to several allergen components, this was not statistically significant after adjusting for age. Children with AHR were 2.5 times more likely to exhibit symptoms than those without AHR. Asthmatic children who were sensitized and had AHR were 6.2 times more likely to have symptoms. The presence of pets in the household did not show any significant connection with the prevalence of AHR.

### 3.3 Asthma symptoms

Based on SPT and IgE component results, sensitization to furry animal components was more frequent among asthmatic children than among controls (Table 3). Asthmatic children sensitized to Can f 1, Can f 6, or both more often reported asthma‐like symptoms than unsensitized children (*p* = 0.002 and *p* < 0.001, respectively) or children sensitized to other furry animal IgE components. Blood eosinophilia was associated with wheeze (*p* = 0.028), but not with other respiratory symptoms. However, sensitization to any furry animal allergen component was a risk factor for blood eosinophilia (Figure 3).

### 3.4 Household pets

Having a pet dog was associated with lower IgE‐sensitization to lipocalin components (Can f 1, Equ c1, and Fel d 4, Table 3). This effect was more pronounced among non‐asthmatic controls. Children with a dog in the same household were also less symptomatic with 0.5 OR (CI 95% 0.3:0.8) and had less frequent blood eosinophilia with OR 0.4 (CI 95% 0.2:0.7) than those without a dog. This effect was more apparent among asthmatic children. This association was not found in controls. Having a dog did not affect the risk of abnormal lung function measures.

### 3.5 SPT

Furry animal allergen component sensitization were significantly associated with SPT results (Table 2). Sensitivity and specificity for dogs were 91% and 87%, for cats was 95% and 97%, and for horses were 100% and 96% respectively (*p* < 0.001). SPT results did not correlate with positive BDT and AHR to methacholine or baseline IOS or spirometry values. However, a strong connection between SPT sensitization to all furry animals and FeNO could be found (*p* < 0.001), however.

## 4 DISCUSSION

This study was based on a variety of objective measures that allow a thorough analysis of the connection between molecule‐specific furry animal sensitization and abnormalities in the respiratory system in childhood. Firstly, we can observe that molecule‐specific IgE‐sensitization to furry animals is a risk factor for bronchial inflammation. Secondly, having a pet dog is associated with a reduced likelihood of developing sensitization to different furry animal species, type 2 bronchial inflammation, and asthma‐like symptoms. Thirdly, sensitization to furry animals is not associated with a positive bronchodilation test after discontinuation of regular asthma medication.

Consistent with a study by Norlund et al.^12^ we showed that sensitization to major allergens such as lipocalins and uteroglobin is associated with blood eosinophilia and asthma symptoms. In our study, serum albumin levels were not connected to asthma‐like symptoms.^10^, ^11^ These findings involving minor allergens would not presumably have been shown or would have been misinterpreted due to cross‐reactivity in previous SPT‐based studies on furry animal sensitization and asthma. The connection between sensitization and blood eosinophilia was also less evident when tested with SPT. In childhood, persistent blood eosinophilia and sensitization to aeroallergens are considered independent predictive factors of chronic asthma later in life.^25^

According to our study, bronchial inflammation measured using FeNO was a more sensitive approach to detect symptomatic individuals and was more connected to sensitization to furry animal components than IOS or spirometry after a discontinuation of anti‐inflammatory asthma medication. It is possible that FeNO only identified patients with allergic symptoms.^26^ However, positive FeNO identified asthmatic children from controls and children with blood eosinophilia from those without asthma better than other lung function measures. The European Respiratory Society's guideline now includes FeNO as a diagnostic measure for childhood asthma and can also be used for asthma phenotyping, predicting exacerbations, and asthma management, especially for allergic asthma.^20^ In atopic individuals, a relatively short pause in anti‐inflammatory asthma medication most likely results primarily in increased eosinophilic bronchial inflammation,^27^ but not abnormal lung function^28^ in atopic children. Longer periods without regular asthma medication may reveal a different outcome.

Although meta‐analyses indicate that owning cats and dogs protects against sensitization to furry animals and childhood asthma,^4^, ^29^ these analyses often use SPTs to establish the prevalence of sensitization and symptoms instead of lung function to diagnose asthma. The standardized extracts used in the SPT tests have shown to have a 20‐fold variation with respect to allergen content.^4^ This is essential as the amount of allergen in the extract and in any exposure in real life correlates with the severity of the allergic reaction. In addition, using the standardized extracts as the only indicator of sensitization may cause overestimation of sensitization due to cross‐reactivity.^5^

Based on our results, having a pet dog was associated with less frequent sensitization to furry animal allergens, asthma‐like symptoms, blood eosinophilia, and bronchial inflammation. In theory, having a dog could normalize the owner's immune reaction patterns due to increased exposure to diverse microbiomes during walks or function as a vector between outdoor and indoor microbiomes, potentially explaining the protective effect.^30^ For patients with an established allergy to furry animals or asthma, avoidance is still the current recommendation for symptom control.^31^ With a multi‐origin disease that changes through time, clinicians cannot predict that having or not having a pet will protect against or cause asthma at the individual level.

AHR is associated with sensitization to furry animals.^7^, ^10^, ^11^ We could not reproduce these findings, most likely because they were derived mainly from adult studies, were performed using SPT, and were not adjusted for confounding factors such as age.^13^ The asymptomatic control group had a higher AHR prevalence than expected, while sensitized asthmatic children with AHR had more lower airway symptoms than those without. Thus, AHR may be indicative of a person's characteristic response to airway irritation present from an early age onwards and leading to a more severe symptom load only later. In a longitudinal study of a birth cohort,^32^ individuals with lower lung function were more frequently sensitized to furry animals at an early age and more often had AHR in school‐age, continuing to exhibit reduced lung function into adolescence. Causality remains unclear, as those with initially higher lung function may have been less predisposed to sensitization and AHR. Our findings partially support this reversed causality, as healthy controls were less often sensitized to furry animals than individuals initially diagnosed with asthma based on abnormal lung function.

There are always limitations to studies involving household pets, as randomized controlled studies are ethically impossible, and the age of initial exposure may be relevant.^29^ Combining several small studies might have exposed the results to heterogeneity and a longitudinal setting would have been more informative for sensitization and asthma.^32^ Nevertheless, our setting allowed us to study the topic with variable objective measures. The reliability of our findings is further supported by the fact that the asthmatic children were originally diagnosed using objective lung function measurements. In addition, all results were carefully adjusted for covariates. The main focus in the present study is in the group of asthma‐like symptoms within which all main endpoints in this study have been analyzed; the controls serve mainly as a demographic comparison of sensitization.

In conclusion, sensitization to molecule‐specific furry animal allergens in children is associated with blood eosinophilia, airway inflammation, and asthma‐like symptoms but not with abnormal lung function. In childhood, sensitization to aeroallergens and blood eosinophilia may predict asthma in later life. Therefore, the possibility that owning a dog is associated with a lower risk of both and asthma‐like symptoms might be worth rewarding that dog with a treat.

## AUTHOR CONTRIBUTIONS

**Katariina Lajunen**: Data curation (equal); formal analysis (equal); funding acquisition (equal); investigation (equal); methodology (equal); software (equal); validation (equal); visualization (equal); writing—original draft (equal). **Anette M. Määttä**: Data curation (equal); funding acquisition (equal); investigation (equal); writing—review and editing (equal). **Kristiina Malmström**: Conceptualization (equal); data curation (equal); investigation (equal); methodology (equal); project administration (equal); supervision (equal); writing—review and editing (equal). **Satu Kalliola**: Conceptualization (equal); data curation (equal); investigation (equal); methodology (equal); project administration (equal); resources (equal); supervision (equal); validation (equal); writing—review and editing (equal). **Hanna Knihtilä**: Data curation (equal); investigation (equal); methodology (equal); resources (equal); validation (equal); writing—review and editing (equal). **Terhi Savinko**: Investigation (equal); methodology (equal); writing—review and editing (equal). **L. Pekka Malmberg**: Conceptualization (equal); investigation (equal); methodology (equal); resources (equal); supervision (equal); validation (equal); writing—review and editing (equal). **Anna S. Pelkonen**: Conceptualization (equal); funding acquisition (equal); methodology (equal); project administration (equal); resources (equal); supervision (equal); validation (equal); writing—review and editing (equal). **Mika J. Mäkelä**: Conceptualization (equal); funding acquisition (equal); methodology (equal); project administration (equal); resources (equal); supervision (equal); validation (equal); writing—review and editing (equal).

## CONFLICT OF INTEREST STATEMENT

The authors declare no conflicts of interest.

## Supporting information

Supplementary Material

## Data Availability

The data that support the findings of this study are available from the corresponding author upon reasonable request.
