# Supplementary material for: Pets, furry animal allergen components, and asthma in childhood
Source: Clin Transl Allergy. 2024 Feb 5;14(2):e12337. doi: 10.1002/clt2.12337 (PMC10839625; doi:10.1002/clt2.12337)
Supplement: Supplementary file 1 — Supplementary Material [file CLT2-14-e12337-s001.docx]

SUPPLEMENTARY INFORMATION (S1)

POWER CALCULATIONS

For children without asthma, 38% test positive for sensitivity to at least one IgE component of a cat, dog, or horse, while among asthmatics, this proportion is 72%. (Patelis 2012 JACI) To achieve a 95% confidence level for a significant result (*P*-value<0.05) with 80% power when comparing asthmatic children with symptom-free controls a cohort of 30 asthmatic children is required.

n= [p1x(100-p1) + p2x(100-p2)]/ (p2-p1)^2^ x f(α,β) =

[38x(100-38) + 72x(100-72)]/ (72-38)^2^ x 7,9 = 30

In the Finnish population, children with asthma-like symptoms include 12% without asthma diagnosis (Lasten allergiset sairaudet 2008). Among them, 60% are sensitized to at least one allergy component, while only 27% of symptom-free controls exhibit sensitivity (Asarnoj 2015 JACI). To achieve a *P*-value under 0.05 when comparing of symptomatic controls to symptom-free controls, a minimum cohort size of 32 children is needed for both groups.

[27x(100-27) + 60x(100-60)]/ (60-27)^2^ x 7,9 = 32

Hence, we need a total of 32 controls and 30+32=62 asthmatic children. This combination is necessary, because some among the asthmatic group may exhibit asthma-like symptoms without actually having the condition. This sample size ensures a significant result with 95% confidence, when accepting a beta cut-off of 20%.

1. Patelis A, Gunnbjörnsdottil M, Malinoschi A, et al. Population-based study of multiplexed IgE sensitization in relation to asthma, exhaled nitric oxide, and bronchial responsiveness. J Allergy Clin Immunol. 2012: 130:397-402
2. Kaila M, Korppi M, Mäkelä M, Pelkonen A, Valovirta E. Lasten allergiset sairaudet. Suomen lastenlääkäriyhdistys, Allergiajaosto. Gummerus kirjapaino Oy, Jyväskylä 2008, 317 p.
3. Asarnoj A, Hamsten C, Wadén K, et al. Sensitization to cat and dog allergen molecules in childhood and prediction of symptoms of cat and dog allergy in adolescence: A BAMSE/MeDALL study. J Allergy Clin Immunol. 2016; 137:813-821
